# Supplementary material for: Co3Gd4 Cage as Magnetic Refrigerant and Co3Dy3 Cage Showing Slow Relaxation of Magnetisation
Source: Molecules. 2022 Feb 8;27(3):1130. doi: 10.3390/molecules27031130 (PMC8840112; doi:10.3390/molecules27031130)
Supplement: Supplementary file 1 [file molecules-27-01130-s001.zip › Supporting information.pdf]

Supporting information for

Co_3Gd_4 cage as magnetic refrigerant and Co_3Dy_3 cage showing slow relaxation of magnetisation

Javeed Ahmad Sheikh*, Himanshu Sekhar Jena and Sanjit Konar

Experimental Section

X-ray Crystallography

Synthesis

[$Co_3Gd_4(\mu_3\text{-}OH)_2(CO_3)(O_2C^tBu)_{11}(teaH)_3$]·$5H_2O$ (1)

[$Co_2(\mu\text{-}OH_2)(O_2C^tBu)_4$]·$(HO_2C^tBu)_4$, **$Co_2$** (100mg, 0.1mmol) was dissolved in CH_3CN (8mL) and triethanolamine, $teaH_3$ (22mg, 0.15mmol) was added to the solution while stirring followed by the addition of $Gd(NO_3)_3.6H_2O$ (45 mg, 0.1mmol) and final mixture was further stirred for ~12 hrs at room temperature. The solution was filtered and the filtrate was kept at 4^0C for slow evaporation. Within 3-4 days purple block shaped single crystals suitable for X-ray analysis were formed. The crystals were collected by filtration, washed with cold CH_3CN and dried in air. Yield, 43% based on **Co_2.** Elemental analysis: Calcd.(found) for $C_{74}H_{136}Co_3Gd_4N_3O_{44}$: C, 34.48(34.51); H, 5.31(5.34); N, 1.63(1.67). Selected IR data (KBr pellet): 3404 (b), 2952 (s), 1607 (s), 1474 (s), 1412 (m), 1374 (w), 1217 (s), 1123.5 (w), 883 (w), 572 (b) cm^{-1}.

[$Co_3Dy_3(\mu_3\text{-}OH)_4(O_2C^tBu)_6(teaH)_3$]$(NO_3)_2$·$H_2O$ (2)

1	2

The same procedure as that of **1** was used for the synthesis of this compound except that $Dy(NO_3)_3.6H_2O$ (45 mg, 0.1mmol) was added instead of $Gd(NO_3)_3.6H_2O$ Yield, 47% based on **Co_2.** Elemental analysis: Calcd.(found) for $C_{48}H_{88}Co_3Dy_3N_5O_{35}$: C, 29.42(29.45); H, 4.52(4.54); N, 3.57(3.59). Selected IR data (KBr pellet): 3438.7 (b), 2961.7 (s), 2075.2 (b), 1560.3 (w), 1484 (s), 1410.5 (m), 1227.9 (s), 1103.5 (m), 923.9 (w), 558.7 (b) cm^{-1}.

Co site	BVS (Assigned oxidation state)	Co site	BVS (Assigned oxidation state)
Co1	2.86(3)	Co1	2.86(3)
Co2	2.83(3)	Co2	2.83(3)
Co3	2.88(3)	Co3	2.82(3)

Table S1. BVS calculations for complexes **1** and **2.**

Table S2. Crystal Data and Structure Refinement for Complexes **1** and **2**

	**1**	**2**
Formula	$C_{74}H_{136}Co_3Gd_4N_3O_{44}$	$C_{48}H_{88}Co_3Dy_3N_5O_{35}$
Formula weight	2574.05	1951.50
T (K)	110(2)	110(2)
Wavelength (Å)	0.71073	0.71073
Space group	*P*-1	*P*21/c
Crystal system	Triclinic	Monoclinic
a/Å	15.552(5)	30.554(17)
b/Å	16.373(6)	19.234(10)
c/Å	25.100(9)	30.327(17)
α/deg	77.745(2)	90.00
β/deg	89.127(2)	115.544(2)
γ/deg	65.270(2)	90.00
V/Å^3	5653(3)	16080(4)
Z	2	4
D_{calcd} (g cm^{-3})	1.512	1.612
μ (mm^{-1})	2.818	3.435
F(000)	2576	7728
θ_{min}, θ_{max} (deg)	2.67, 25.00	2.19, 25.00
Reflection collected	19227	28319
unique reflections	19911	26869
R_1, wR_2 ($I \geq 2\sigma(I)$)	0.0433, 0.1273	0.0760, 0.2181
Goodness of fit (GOF) on F^2	0.902	0.927

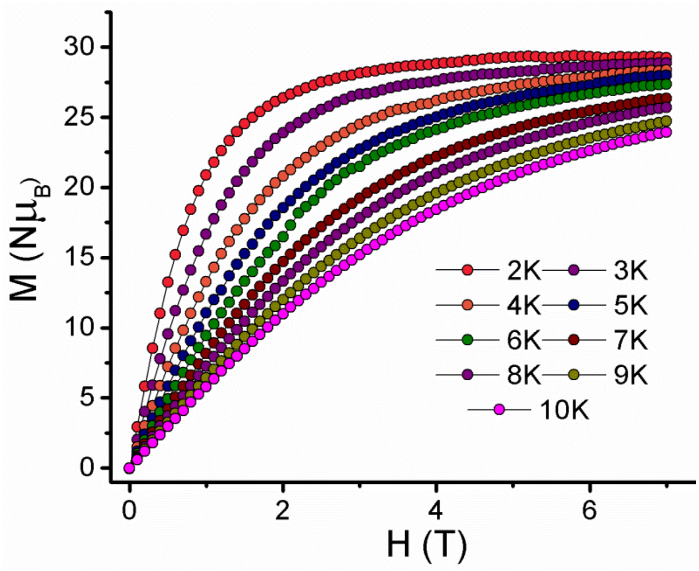

Figure S1 Field-dependencies of isothermal normalized magnetizations for complex **1** collected for temperatures ranging from 2-10 K

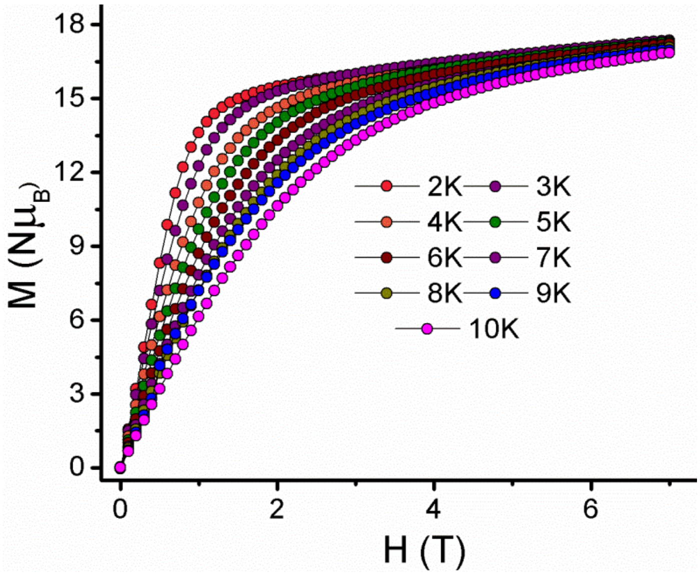

Figure S2 Field-dependencies of isothermal normalized magnetizations for complex **2** collected for temperatures ranging from 2-10 K.

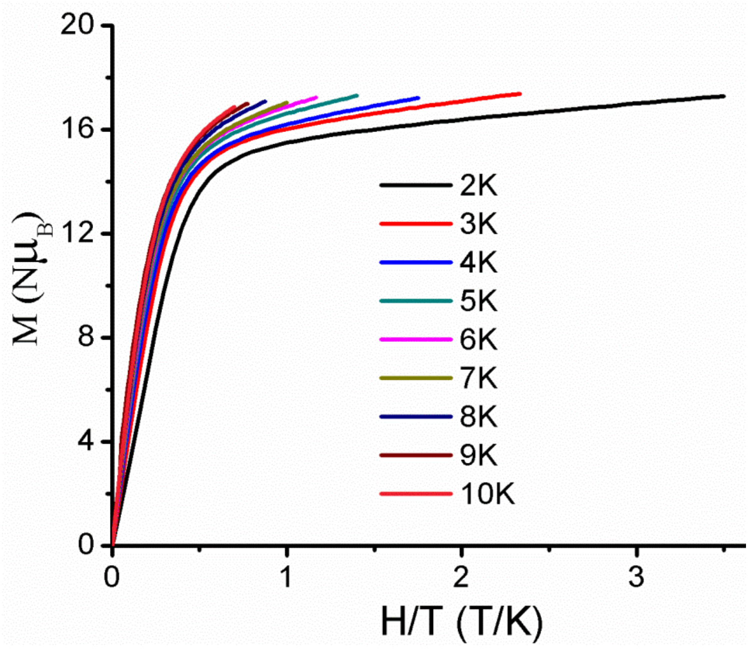

Figure S3 M/Nμ$_B$ vs H/T plots for complex **2** at 2-10 K.

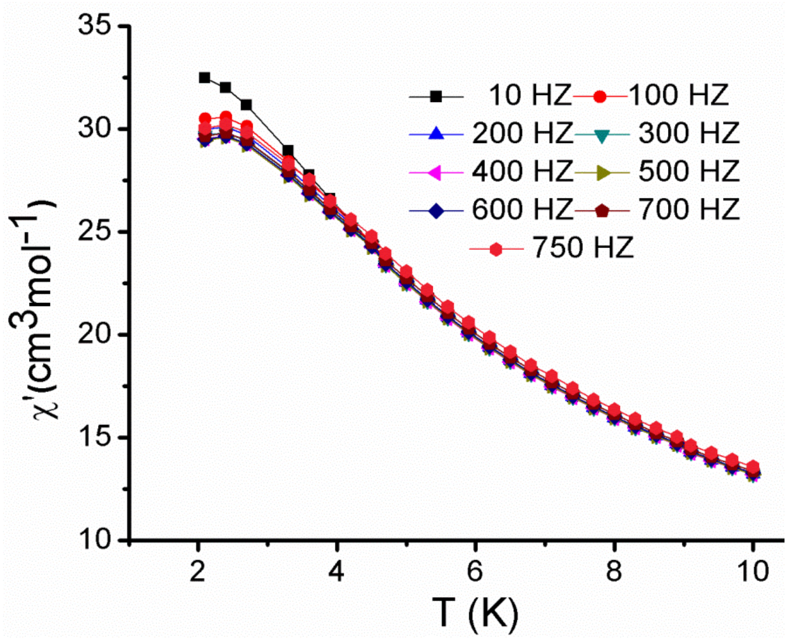

Figure S4 Temperature dependence of the in-phase (*χ′*) ac susceptibility for complex **2** under zero dc field.

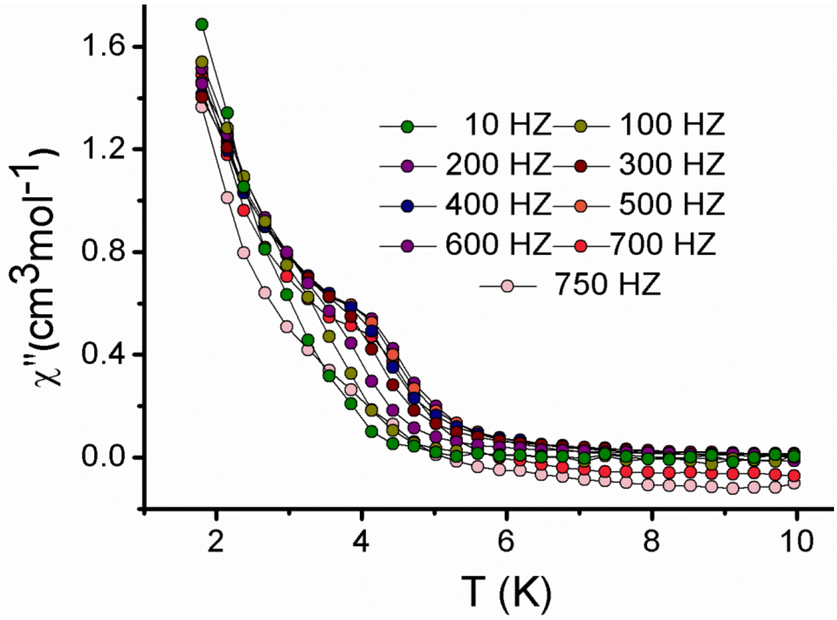

Figure S5 Temperature dependence of the out of phase (χ'') ac susceptibility for complex **2** under zero dc field.

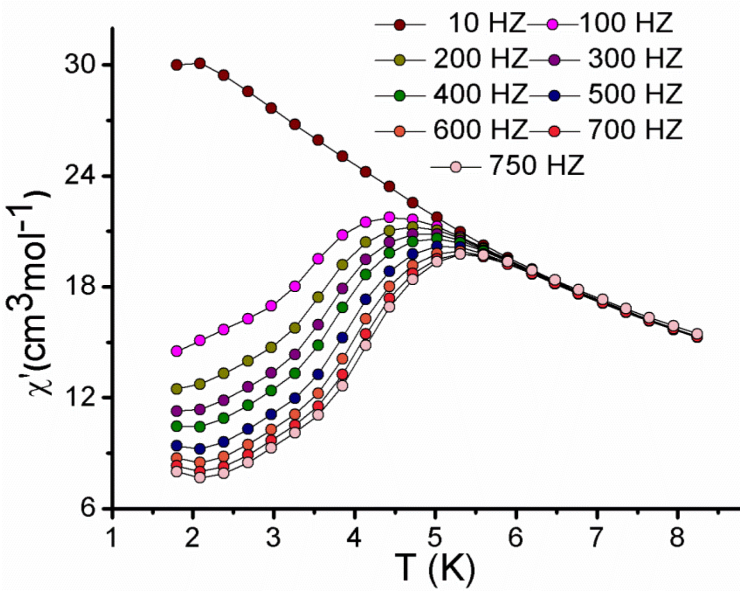

Figure S6 Temperature dependence of the in-phase (χ') ac susceptibility for complex **2** under 2000 Oe dc field.

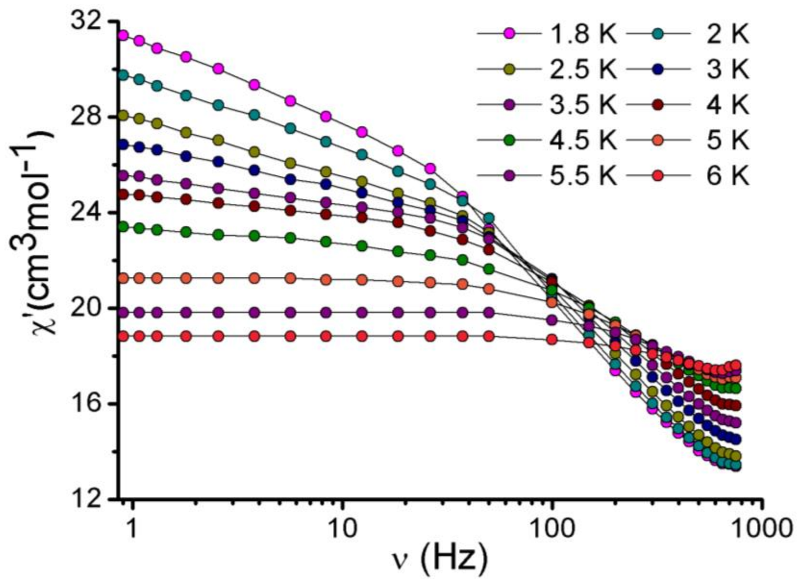

Figure S7 Frequency dependence of the in-phase (χ') ac susceptibility for complex **2** under 2000 Oe dc field.

References:

1 *SAINT*, Program for Reduction of Area Detector Data, **V6. 63**; BRUKER AXS Inc. Madison, WI.

2. Sheldrick, G. SADABS, Program for Absorption Correction of Area Detector Frames; BRUKER AXS Inc.: Madison, WI.

3. Sheldrick, G. *SHELXL-97, Program for Crystal Structure Refinement*; University of Gottingen: Gottingen, Germany, 1997.

4. PLATON: A. L. Speck, *J. Appl. Crystallogr.* 2003, **36**, 7.

5. A. L. Speck, PLATON. Molecular Geometry Program. *J. Appl. Crystallogr*. 2003, **45**, 1.

6. O. Kahn, *Molecular Magnetism*; Wiley-VCH: New York, 1993.
